# Opioids and Vitamin C: Known Interactions and Potential for Redox-Signaling Crosstalk

**DOI:** 10.3390/antiox11071267

**Published:** 2022-06-27

**Authors:** Mackenzie Newman, Heather Connery, Jonathan Boyd

**Affiliations:** 1 Department of Orthopaedics, School of Medicine, West Virginia University, Morgantown, WV 26506, USA; msnewman@mix.wvu.edu (M.N.); hc00034@mix.wvu.edu (H.C.); 2 Department of Physiology and Pharmacology, Robert C. Byrd Health Sciences Center, West Virginia University, Morgantown, WV 26505, USA

**Keywords:** vitamin C, opioids, mu opioid receptor, oxidative stress, signaling, metabolism, crosstalk

## Abstract

Opioids are among the most widely used classes of pharmacologically active compounds both clinically and recreationally. Beyond their analgesic efficacy via μ opioid receptor (MOR) agonism, a prominent side effect is central respiratory depression, leading to systemic hypoxia and free radical generation. Vitamin C (ascorbic acid; AA) is an essential antioxidant vitamin and is involved in the recycling of redox cofactors associated with inflammation. While AA has been shown to reduce some of the negative side effects of opioids, the underlying mechanisms have not been explored. The present review seeks to provide a signaling framework under which MOR activation and AA may interact. AA can directly quench reactive oxygen and nitrogen species induced by opioids, yet this activity alone does not sufficiently describe observations. Downstream of MOR activation, confounding effects from AA with STAT3, HIF1α, and NF-κB have the potential to block production of antioxidant proteins such as nitric oxide synthase and superoxide dismutase. Further mechanistic research is necessary to understand the underlying signaling crosstalk of MOR activation and AA in the amelioration of the negative, potentially fatal side effects of opioids.

## 1. Introduction

Opioids are among the most widely used classes of pharmacologically active compounds both clinically and recreationally. While opioids have been invaluable in the clinic for analgesia, they produce numerous side effects and are highly addictive [1,2,3,4]. Their principal binding at μ opioid receptors (MOR) causes potentially fatal opioid induced respiratory depression (OIRD) by inactivation of the pre-Bötzinger complex [5], leading to decreased breathing, systemic hypoxia, oxidative stress, and free radical generation. Ascorbic acid (AA; vitamin C), the antioxidant vitamin at the highest circulating concentration in the human body (compared to vitamins A, D, E, and derivatives) [6,7], has critical roles in processes such as neurotransmission [8,9], wound healing [10], and immune homeostasis [11] and may be able to quench aberrant oxidative signaling associated with opioid exposures. The low toxicity of vitamin C makes it an ideal investigational adjuvant to address some of the negative effects of opioid administration. This review seeks to address the current state of in vitro and in vivo co-administration research associated with opioids and vitamin C, while identifying potential underlying mechanisms of interactions. Additionally, this review identifies current gaps in the literature associated with intracellular redox-signaling mechanisms between vitamin C and opioids and presents focused paths forward for research in the field.

The role of vitamin C on opioid administration, specifically morphine as the classical opioid, has been briefly investigated both clinically and in model systems. Few studies have been carried out in humans, but some small cohorts have shown reduced circulating vitamin C levels in chronic opioid users [12,13]. Treatment with vitamin C has been shown to decrease opioid withdrawal symptoms in guinea pigs [14], rats [15], and humans [16,17]. AA has been described as a solubilizing agent for street heroin base, although the amount of AA required is far below the amounts used in laboratory and clinical research settings [18,19]. In post-operative humans, administration of vitamin C has been shown to lower morphine consumption [20], nausea, and overall pain scores [17,21,22]. The ability of vitamin C to potentiate analgesia from morphine has been noted in humans [17,23,24], as well as mice [25,26] and rats [27], measured most often via tail-flick or hot plate test. Overall, brain concentrations of AA have been shown to be increased after morphine administration in rats [28,29,30], but effects in other organ systems are currently unknown. While most studies on the effects of vitamin C and morphine (or similar opioids) in humans are focused on changes in withdrawal symptoms in abuse settings and post-surgery recovery outcomes, much work is left to be done to understand the potential critical interaction mechanisms that may lead to these integrated physiological responses.

## 2. Opioid Activity and Metabolism

**Figure 1:**
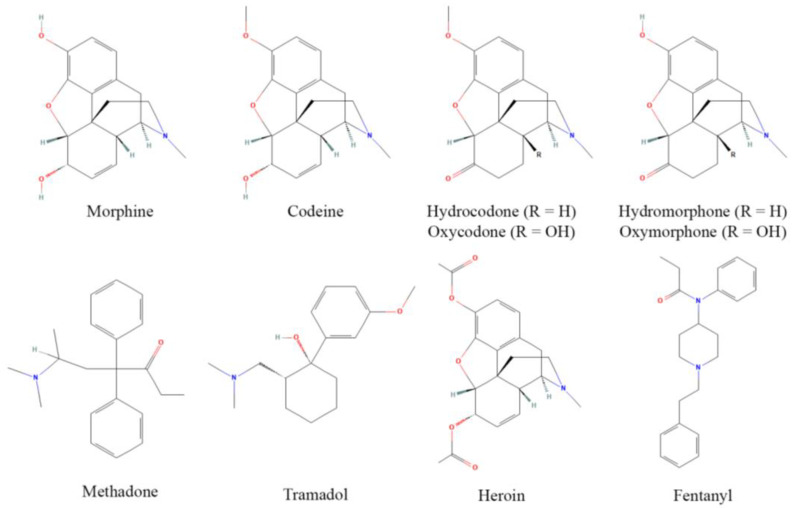
Structures of common opioids. Some prominent clinical opioids include morphine, codeine, hydrocodone, oxycodone, hydromorphone, oxymorphone, methadone, and tramadol, while heroin and fentanyl are most often subject to illicit use. Structures are derived from [35].

Opioids, originally derived from the opium poppy plant, are one of the largest and most potent classes of pharmacologically active compounds, and are readily used for their antinociceptive properties, although they are often abused due to their euphoric effects at higher doses. The discovery of MOR and endogenous opioid peptides in the 1970s [31] has led to a revolution in opioid design, discovery, and implementation [32]. Commonly used opioids include prescription drugs such as morphine, codeine, hydrocodone, oxycodone, hydromorphone, oxymorphone, methadone, and tramadol, as well as heroin and fentanyl, which have major roles as drugs of abuse (although fentanyl is still used clinically) (Figure 1). These act as either partial or full agonists of the MOR, the focal receptor for antinociception and analgesia, and principally bind surface opioid receptors [33]. Opioids frequently have partial affinity for κ and δ opioid receptors as well, yet analgesic contributions from these receptors are contested [34].

Opioid receptors are present in nearly all tissues and cell types [36,37], and are especially upregulated in the nervous system, but also have critical roles in the gastrointestinal tract, respiratory tract, and cardiovascular system where they modulate inflammatory signaling [38]. MOR is a typical G-protein-coupled receptor (GPCR), acting via an intracellular inhibitory G protein cascade (G_i_/G_o_), preferentially through G_o_ and G_i2_, but also G_i1_, G_i3_, G_z_, and G_16_ [39]. After ligand binding, dissociation of the G-protein complex inhibits adenylate cyclase (AC) activity, causing decreased intracellular levels of cyclic adenosine monophosphate (cAMP) [26] and downstream reductions in protein kinase A activity, as well as activation of the mitogen-activated protein kinase (MAPK) cascade; these actions cause CREB translocation to the nucleus to alter transcription, including redox-relevant genes encoding for neuronal nitric oxide synthase (nNOS) [40], NAD(P)H oxidases, catalase (CAT), and superoxide dismutase (SOD) [41]. While the Gα complex activates phospholipase C, generating inositol-3-phosphate (IP3) and thus inducing intracellular calcium release from the endoplasmic reticulum, the Gβ/γ complex blocks plasma membrane calcium channels, leading to an overall increase in intracellular calcium signaling [39,42].

**Figure 2:**
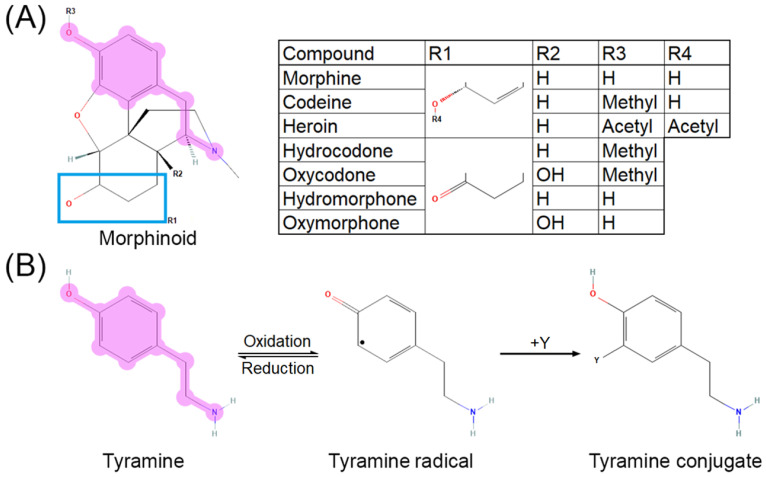
Tyramine moiety in morphinoid pharmacophore. (**A**) Morphinoid pharmacophore with tyramine moiety highlighted. (**B**) Reaction mechanism for oxidation of tyramine (adapted with permission from Ref. [56]. 2013, American Chemical Society). Reversible oxidation of tyramine (**left**) leads to a neutral, reactive radical intermediate species (**middle**) which can quench free radicals such as superoxide, hydroxide radical, nitrite radical “Y”, yielding a substituted phenol (**right**). Reaction of tyrosine, a tyramine-containing amino acid, with the nitrite radical or peroxynitrite forms nitrotyrosine, a residue disruptive to protein–protein interactions.

The cytochrome P450 system is primarily responsible for most opioid metabolism, through CYP3A4 and CYP2D6, for species such as codeine, hydrocodone, oxycodone, fentanyl, methadone, and tramadol [43]. Morphine, however, does not go through first-pass metabolism and is instead directly glucuronidated by UGT2B7 into morphine-3-glucuronide or morphine-6-glucuronide [43,44]. Its major metabolites, morphine-3-glucuronide and morphine-6-glucuronide, are active at MOR. Major metabolism occurs in the liver, heart, and kidneys. While UGT2B7 has not been described to promote free radical formation as reactive oxygen species (ROS) or reactive nitrogen species (RNS), CYP3A4 and CYP2D6 are established as contributing to ROS generation [45]. These CYP enzymes are notably responsible for the majority of all drug metabolism [46]. Opioid metabolism is confounded by pharmacokinetic and pharmacodynamic interactions that warrant consideration for signaling transduction research. For example, morphine-6-gluconuride has a higher affinity for MOR than morphine [47], yet cannot pass through the blood–brain barrier as efficiently due to higher polarity [48]. O-demethylation of the anisole group in oxycodone to oxymorphone, hydrocodone to hydromorphone, and codeine to morphine via CYP2D6 [49,50], as well as deacetylation of the phenylacetate in heroin to 6-monoacetylmorphine by esterases [51,52], exposes a tyramine-like phenol moiety (Figure 2A) that can undergo oxidative transformation to quench free radicals [53,54] via a neutral radical intermediate [55] through the mechanism shown in Figure 2B (adapted from [56]). Common polymorphisms in CYP2D6 lead to decreased enzyme activity and therefore decreased conversion of the aforementioned opioids to their active metabolites [57,58], thus potentially shunting the parent compounds toward alternative metabolic pathways. Morphinone derivatives can also undergo secondary elimination via conjugation to glutathione, although the MOR affinities of these metabolites have not been characterized [59,60].

## 3. Vitamin C Activity and Metabolism

Vitamin C is a well-known antioxidant that helps protect cells against the detrimental effects of free radicals such as ROS and RNS [61,62,63]. Vitamin C is synthesized in plants and protists from galactose and from gulonolactone in many vertebrates (e.g., mice, rats, rabbits, cats, dogs, and sheep), yet humans, primates, bats, guinea pigs, and some bony fish (notably zebrafish) possess pseudogenes or entirely lack the gene encoding for L-gulonolactone oxidase (*Gulo*), the enzyme required to produce it [64,65]. *Gulo* knockout mice (derived on a C57BL/6 background) have been established to investigate dietary AA-dependent processes [66,67,68], yet *Gulo* has not been ablated in other vitamin C-synthesizing mammals, such as rats and rabbits.

At physiological pH, AA exists as its conjugate base and prominently serves as a direct antioxidant via ascorbyl radical [69] or as an enzyme cofactor. Vitamin C is well-tolerated in humans with zero reported deaths; its LD50 is estimated around 3.3 g/kg–11.9 g/kg in rodents [70]. The low toxicity of AA is attributable to high water solubility, allowing rapid clearance upon administration; however, excess vitamin C can result in oxalate production, thus administration is contraindicated for individuals with kidney stones [71]. AA functions in numerous hydroxylation reactions, often Fe^2+^-dependent, such as oxidation of lysine and proline residues in collagen to form mature fibrils, DNA repair, and activation of hypoxia-induced factors (e.g., HIF1α) [72]. It can function as a pro-oxidant species [73], yet this biological role has not been fully established. The oxidized form of AA, dehydroascorbic acid (DHA), can be recycled back into vitamin C at the expense of NADPH or glutathione [74].

The underlying mechanisms of vitamin C transport, under normal conditions or oxidative stress, have not been fully elucidated. While import of AA primarily occurs via SVCT1 and SVCT2, two sodium-dependent cotransporters, DHA is imported through GLUT transporters and recycled into vitamin C [75]. It is important to note that GLUT-mediated transport of DHA is competitively inhibited by glucose as they share the same transporter [76]. Vitamin C is absorbed in the intestine through a saturable, dose-dependent active transport process, where it is converted to DHA and then reduced back into AA once it has entered epithelial tissue/cells. Reabsorbed excess vitamin C travels through the renal tubules and is excreted in the urine [77].

Efflux mechanisms have yet to be determined, but are unlikely to be a function of simple diffusion despite a high intracellular:extracellular concentration gradient caused by the polarity and hydrophilicity of AA. DHA is structurally unstable in physiological conditions [78] and may be further hydrolyzed to diketogulonic acid [79], which can then be decomposed to metabolites such as oxalic acid [80]. Efflux may occur as a function of membrane disruption resulting from oxidative stress, and it has been hypothesized that transport-mediated cellular swelling may activate volume sensitive anion channels in the basolateral membrane thus enabling the export of AA [81]. Vitamin C levels after morphine administration have been noted to be higher in the striatum, but not nucleus accumbens in one study [82], yet higher in the nucleus accumbens than the striatum in another [83], suggesting that cell-specific efflux mechanisms may be present.

Vitamin C acts as a cofactor in various metabolic reactions, including iron metabolism [78], neuronal energy metabolism [84], and lipid metabolism [85]. Vitamin C is both a major electron donor directly to Fe^2+^ and also modulates iron metabolism through stimulation of ferritin synthesis, which inhibits ferritin degradation by lysosomes and reduces efflux of cellular iron [78]. Moreover, vitamin C also facilitates the transformation of cholesterol into bile acids through modulation of microsomal 7α-hydroxylation catabolism of cholesterol in the liver, and increases the rate of hydroxylation reactions through the maintenance of metal ions in their reduced state to optimize hydroxylase and oxygenase function [86]. In the brain, DHA taken up by neurons inhibits glycolysis, activates the pentose phosphate pathway which produces NADPH, oxidizes glutathione, and stimulates lactate production and uptake in the neurons [84].

## 4. Opioids, Vitamin C, and Neurotransmission

When morphine or other agonists bind to MOR, the mesolimbic reward system is activated. GABA interneurons in the ventral tegmental area (VTA), more specifically the rostromedial tegmental nucleus, are inhibited, leading to a disinhibition of dopaminergic neurons, causing release of dopamine to the nucleus accumbens to produce feelings of euphoria [87]. GABA input may also be inhibited in the nucleus accumbens itself, as well as in the periaqueductal gray and raphe magnus, where this affect is thought to contribute to the analgesia that occurs with opioid use [34]. In concert with VTA dopamine release, suppression of norepinephrinergic neurons in the locus ceruleus (LC) by MOR activation reduces wakefulness, blood pressure, and respiration [87]. However, the deleterious respiratory effects of opioids are primarily attributed to the high expression of MOR in the pre-Bötzinger complex, a region of the ventral medulla [88,89], which is thought to generate respiratory rhythm [90].

MOR desensitization is focal in cases of opioid tolerance, addiction, and withdrawal. This involves adaptation of dopamine release from the VTA and norepinephrine from the LC, producing hyperresponsivesness to pain and elevated alertness [34,87]. Mechanistically, MOR typically functions first through G_i/o_ to signal with temporal cessation after β-arrestin recruitment to the receptor [34,91]. With tolerance, signaling becomes β-arrestin-biased, reducing antinociception and producing enhanced respiratory depression [34,87,92,93]. Research into biased MOR signaling is ongoing, with the potential to severely reduce the negative central effects of opioids [94,95,96].

Whereas opioids primarily compete for endogenous opioid peptide binding sites on μ, κ, and δ receptors [97], vitamin C has broader activity in neurotransmission. Neurons and glia both rely heavily on a tightly regulated surplus of vitamin C (millimolar levels) via uptake through the glucose transport system and sodium-coupled active transport [79]. GLUT-1 transporters are primarily responsible for vitamin C delivery across the blood–brain barrier in the form of DHA [98]. Once DHA has been recycled to retained AA, subsequent release can be triggered by glutamate uptake in astrocytes, which has been proposed to cause extracellular swelling and allows diffusion of AA through activation of volume-regulated anion channels [99,100].

Vitamin C is known to modulate T-type Ca^2+^ channels, thus modulating neural excitability [9]. Recently, studies have suggested a pivotal role of these channels, particularly Ca_V_3.2 T-type channels, in the processing of pain signals [101]. These channels regulate excitatory neurotransmission, notably glutamate, in peripheral nerve endings of nociceptors. These calcium channels are sensitive to inhibition by divalent metals such as zinc and contain a Zn^2+^ binding histidine residue (His^191^) on domain I. AA selectively suppresses Ca_V_3.2 T-type channels via simultaneous binding of Zn^2+^ and AA at His^191^ [101]. Inactivation of these channels to allow calcium efflux by vitamin C may be nullified by the action of MOR activation to increase intracellular Ca^2+^ concentrations [42], yet this has not been determined experimentally.

AA is not a direct neurotransmitter; however, extracellular AA may act as a modulator of neurotransmission by distinct mechanisms. It can attenuate the activity of NMDA receptors in the forebrain through its various redox reactions [9] as discussed later, and may also play a role in the release of biogenic amines such as dopamine and pituitary neuropeptides [102].

Vitamin C also plays a significant role in the biosynthesis of neurotransmitters and neuropeptides directly involved in opioid-induced analgesia and dependence. As a cofactor in dopamine β-hydroxylase and 4-hydroxyphenylpyruvate dioxygenase, vitamin C is required for the synthesis of dopamine and upstream shunting of tyrosine, a precursor of dopamine, to alternative catabolic pathways. Immediate biotransformation products of tyrosine metabolism, tyramine [103,104,105,106] and L-DOPA [104,105,106,107], as well as further downstream products epinephrine/norepinephrine [108] and homogentisic acid [109] can act as direct antioxidants. AA has been shown to elevate recycling of tetrahydrobiopterin (BH4) [102], which is critical in the synthesis of small neurotransmitters such as dopamine, epinephrine, norepinephrine (via tyrosine hydroxylase; TH), serotonin (via tryptophan hydroxylase; TPH), and nitric oxide (NO, via nitric oxide synthase; NOS) [8]. Morphine administration has been associated with increased TH protein levels in the ventral tegmental area, but without changes in total TH amount in the nucleus accumbens [110,111]; both regions have been implicated in reward-seeking behavior [112]. Morphine has been shown to increase TPH activity in the cerebral cortex, midbrain, and pons-medulla, but not the spinal cord in rats [113]. NOS activity is known to be increased by morphine administration in neuronal and peripheral tissues (discussed later with MAPK activation). Combined, the action of vitamin C (to increase BH4 levels) and opioids (to upregulate enzyme levels and activity) lead to catecholamine, serotonin, and NO synthesis that complicates the redox status of target cells; although these compounds can act as antioxidants, morphine is generally known to increase ROS and RNS [114,115,116].

Beyond additive activity, AA may indirectly antagonize opioid activity through receptor binding competition via endomorphin synthesis. This antagonism is possible due to the ability of AA to maintain stores of Cu^+^ by direct reduction of Cu^2+^. Cu^+^ is involved in the synthesis of numerous small neuropeptides (e.g., endomorphins) as a cofactor in peptidylglycine α-amidating monooxygenase. This enzyme can also catabolize glycyl-fatty acids to produce fatty acid amides [117] with direct and indirect activities on the aforementioned neurotransmitter systems, but also endocannabinoid [118] and vanilloid [119,120] signaling, both of which are implicated in redox balance.

## 5. Opioids, Vitamin C, and Direct Oxidative Stress Modulation

Under normal conditions, diatomic oxygen concentrations are modulated by a series of enzymes that have the capacity to suppress radical production and propagation. These antioxidant enzymes consume glutathione or NADPH stores to reduce downstream DNA, lipid, and protein oxidation, which ultimately prevents mitochondrial and whole-cell damage from ROS and RNS. SOD, in tandem with CAT, and glutathione peroxidase (GPx) are the major endogenous antioxidant defenses that have been studied for both opioids and vitamin C.

As discussed in “Opioid Activity and Metabolism” above, morphinoids can function as direct antioxidants upon oxidative activation [53,54,55]. Despite this activity, morphine and other morphinoid-signaling functions cause overall increases in the production of ROS and RNS, yet the underlying mechanisms causing this have not been fully elucidated. However, the major antioxidant defense enzymes have been studied in response to opioid exposure: SOD activity has been shown to be decreased in human erythrocytes [121], plasma [12], and sperm [122], as well as in rat cerebrum [123], hippocampus [124], and liver [125]; CAT has been shown to be decreased in human erythrocytes [121] and plasma [12], as well as rat cerebrum [123] and livers [125]; and GPx has been shown to be decreased in human plasma [12] and sperm [122], as well as rat whole brain [126], cerebrum [123], and hippocampus [124], after opioid exposure. Few studies have looked at alternate redox enzymes, but have revealed increases in thioredoxin [107] as well as decreases in peroxiredoxin [127] and myeloperoxidase [128].

The effect of opioids on the major antioxidant enzymes above are straightforward, unlike the role of vitamin C. During oxidative stress, vitamin C primarily acts as an antioxidant species (as ascorbyl radical) to directly quench reactive species such as peroxynitrite and singlet oxygen via single-electron reduction [69,129,130]. Changes in antioxidant enzymes and oxidation markers (e.g., SOD, glutathione, and malondialdehyde) are highly cell- and tissue-dependent with regard to vitamin C status. Despite the ability of vitamin C to reduce overall oxidant load, thus lessening the required activity of enzymes such as SOD, CAT, and GPx, studies have shown mixed results. Human red blood cells from healthy, non-smoking volunteers have been shown to have decreased SOD activity after vitamin C supplementation [131]. In rats with vitamin C supplementation, SOD activity has been shown to be decreased in astrocytes under normoxia [132], yet activity is unchanged in red blood cells and liver [133]. In the sera of spontaneous hypertensive rats given a high-salt diet with vitamin C supplementation, SOD activity has been shown to be increased [134]. While SOD activity was not shown to be significantly different from controls in vitamin C supplemented patient sera after an acute experience of repeated diving apnea [135], it was shown to be increased in sera from type 2 diabetics given vitamin C [136], suggesting a potential cumulative adaptive response in SOD from prolonged oxidative stress with vitamin C exposure. In contrast, a small cohort study showed no significant change in SOD activity in the saliva of chronic smokers [137] given vitamin C, yet supplemented rats exposed to chronic cigarette smoke had increased liver SOD activity [138].

The complex story associated with vitamin C and redox-specific enzymes continues with CAT activity, where human red blood cells have been shown to not be affected by vitamin C supplementation [131]. CAT also was not affected in red blood cells or livers of vitamin C supplemented rats in one study [133], yet liver activity was increased in another study of the effects of vitamin C on cigarette smoke-exposed rats [138]. CAT activity has been shown to be elevated in the sera of rats under chronic variable stress [139]. In humans, vitamin C supplementation increased CAT activity in the first 24 h of hydrogen peroxide treatment in lymphocytes of a post-exercise cohort [140], but showed no significant differences from 24 to 48 h. Human serum from individuals exposed to the same diving apnea study mentioned previously showed no significant changes in CAT activity with vitamin C supplementation [135].

The impact on GPx has not been investigated as frequently for its role in oxidant stress with vitamin C supplementation. In normoxic, healthy human red blood cells, vitamin C supplementation has no effect on GPx activity [131]. In normoxic rats, supplementation increased GPx activity in red blood cells, but not in livers [133]. In contrast, after supplementation, GPx activity was unchanged in rat livers following chronic cigarette smoke exposure [141]. It was increased in supplemented type 2 diabetic human sera, however [136]. Malondialdehyde levels, downstream of GPx, were not significantly different in supplemented rats under chronic stress [139], yet were decreased in supplemented humans after acute exercise and in type 2 diabetes [136].

Overall, direct comparison of previous literature associated with vitamin C and antioxidant enzymes (SOD, CAT, and GPx), which were either increased [134,136,138,139,140] or unchanged [135,139,140,141] may be confounded by the samples assayed. In humans, samples in previous research have been limited to saliva and blood. Although these may reflect whole-body oxidative enzyme status, organ-specific effects are critical to define, as conditions such as smoking and type 2 diabetes are designated to have target organs (e.g., lungs and pancreas). Despite two of the human studies involving acute oxidative stress (diving apnea and exercise) and two involving chronic conditions (smoking and type 2 diabetes), the researchers used different samples (i.e., isolated leukocytes and serum for acute condition, and saliva and serum for chronic). Future studies should be standardized to a single type of sample, such as a serum, and address all three antioxidant defense enzymes (SOD, CAT, and GPx).

Rat studies are complicated by differences in sampling as well, but also by the presence of an active rat *Gulo* gene, which may therefore have implications on the pharmacology, particularly transport and retention, of exogenous vitamin C. Across rat models, oxidative-stress-linked condition or disease has either increased SOD and CAT or had no change across SOD, CAT, and GPx. Animal models are critical for vitamin C supplementation research as they allow for direct organ and tissue sampling, yet standardization (e.g., using a specific strain) is critical. *Gulo* knockout mice or rats are better suited for translatable research toward the local effects of vitamin C supplementation.

## 6. MOR Activity and NOS

**Figure 3:**
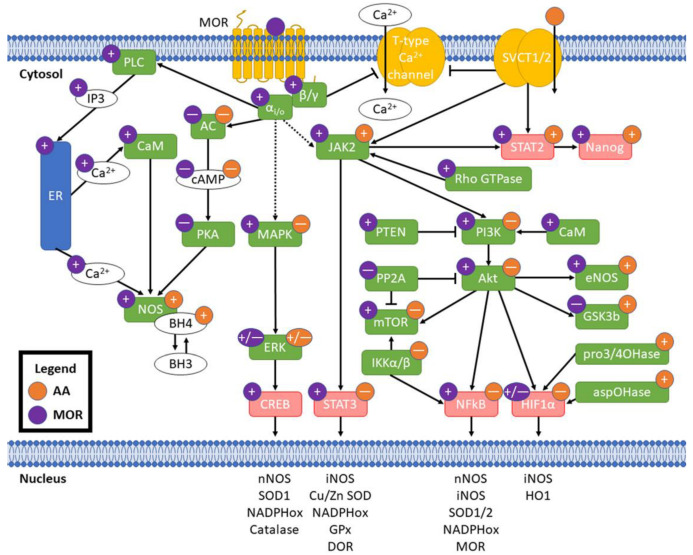
Potential crosstalk between MOR activation and AA. Purple circles indicate effect of MOR activation and orange circles indicate effect of AA; “+” is an increase, “—” is a decrease, and “+/—” indicates mixed effects from literature. Pointed arrowheads indicate activation and flat arrowheads indicate decreased activity. Solid arrows indicate direct effects and dashed arrows indicate indirect/distal effects. Green boxes are enzymes and red are transcription factors. Transcription of example redox genes and proteins listed in the nucleus is affected by their linked transcription factors. MOR: μ opioid receptor; SVCT1/2: sodium-dependent vitamin C transporters 1/2; αi/o: Gαi/o subunit; β/γ: Gβ/γ subunit; PLC: phospholipase C; IP3: inositol 3-phosphate; ER: endoplasmic reticulum; CaM: calmodulin; NOS: nitric oxide synthases; BH3: trihydrobiopterin; BH4: tetrahydrobiopterin; AC: adenylate cyclase; cAMP: cyclic adenosine monophosphate; PKA: protein kinase A; pro3/4OHase: prolyl 3- and prolyl 4-hydroxylases; aspOHase: asparaginyl hydroxylase.

Individual mechanistic interactions between vitamin C and opioids have not been researched previously. Despite this, both AA and opioids have been shown to separately affect signaling pathways at overlapping and discrete nodes with crosstalk potential. These pharmacodynamic interactions may underlie the capacity of vitamin C to ameliorate oxidative stress induced by opioids beyond ablation of free radicals by vitamin C or indirect changes in antioxidant defense enzymes. Pharmacokinetic interactions, e.g., metabolic changes in opioid structures that change ligand affinity to MOR [47] or intracellular redox cofactor-sensitive recycling of DHA to AA [74] should be taken into consideration for experimental validation of potential crosstalk in oxidative stress. A summary of the potential pathway crosstalk described here is provided in Figure 3 and described in detail below.

AA has mixed effects on the inhibitory G protein cascades initiated by MOR. Both MOR and AA lead to inhibition of AC and reduced cAMP levels [26,142,143,144]. MAPK signaling, induced directly from MOR activation, is linked to ROS generation in an ERK/JNK-dependent manner [145]. While AA has been shown to decrease MAPK signaling [146,147], ERK has been shown to be both activated and inhibited by both MOR [148,149] and AA [150,151,152]. Accumulation of β-arrestin at MOR decreases signaling post-activation [91], ceasing this activity. Increased cytosolic Ca^2+^ concentrations, another product of MOR activation [42], leads to NOS activation from direct calmodulin binding to the enzyme [153]. MOR activation has been shown to cause increased endothelial NOS (eNOS) activity and NO release in vascular tissue, potentially via μ3 opioid receptors [154], and has been linked to increased nNOS activity and NO production in neuronal tissue [155]. Morphine agonism on mitochondrial opioid receptors has also been shown to be coupled to calcium-dependent NO production from constitutive cNOS [156].

BH4 is required as a cofactor for many NOS enzymes which are upregulated by opioids. Overall, NOS activity has been shown to be increased by vitamin C [157] via increased BH4 levels. This phenomenon is most likely explained by the ability of vitamin C to recycle BH3, the reduced form of BH4 [158], and thus prevent BH3 from further catabolism. The additive effect of opioids and AA to promote NOS activity confers elevated NO, which can outcompete superoxide at SOD in a concentration-dependent manner [159]. NO can also combine with superoxide to form peroxynitrite (ONOO-), which has a longer half-life than superoxide [160]. Increased NOS activity in tandem with decreased SOD activity (and therefore elevated free superoxide) from morphine is likely to cause increased ONOO- production, but this has not yet been experimentally determined. Although ONOO- is difficult to measure directly in biological systems, its predominant marker, nitrotyrosine, has been shown to be elevated after morphine treatment [161]. A general scheme for nitrotyrosine formation is given in the section “Opioid Activity and Metabolism”, Figure 2B, where “Y” is either nitrite radical or ONOO-. This modified amino acid can disrupt protein–protein signaling at tyrosine residues directly and impact local binding environment due to pKa shift at the phenol moiety [162]. In contrast, vitamin C reduces the formation of ONOO- via mechanisms such as reductions in NADPH oxidase activity [163]. The degree to which the combination of vitamin C and morphine co-administration affects ONOO- levels has not yet been explored.

## 7. Vitamin C and MOR: Potential for Crosstalk

JAK/STAT signaling is crucial in cytokine-induced transcriptional regulation resulting from many cellular processes, particularly those involved in immunostasis, and can be activated by ROS generation [164]. The canonical pathway begins with ligand binding to a cytokine receptor, causing receptor dimerization and phosphorylation of the receptor by JAK1, JAK2, or JAK3. These phosphorylation sites recruit STAT proteins, which, after dimerization and phosphorylation, translocate to the nucleus to alter transcription. Opioid receptors have not been shown to bind latent JAK proteins. However, morphine has been shown to induce JAK2 phosphorylation with no change in JAK1 in reperfusion-ischemia-injured rat hearts [165]. The ability of MOR activation to induce JAK phosphorylation may be attributable to downstream rho GTPase activity [164]. Uptake of vitamin C through SVCT2 has been shown to be induced by phosphorylation of the C-terminus of SVCT2 by JAK2 [166], therefore suggesting a localized additive effect between morphine and vitamin C; both compounds have also been shown to cause downstream STAT2 phosphorylation [165,166] resulting in upregulation of Nanog, a transcription factor noted in pluripotency and cancer. JAK2 is also capable of phosphorylating STAT3; while studies on morphine have shown increased activation [165,167], vitamin C abrogates STAT3 activation [168,169] potentially by quenching activation from ROS. Furthermore, activated STAT3 has been shown to regulate iNOS [170,171], NADPH oxidases, GPx, SOD3, and OPRD1 [172], which directly encodes for the δ opioid receptor (DOR).

JAK2 activation by morphine has also been described to upregulate the phosphoinositol-3-kinase (PI3K)/Akt (protein kinase B) pathway [165,173]. This cycle-regulating pathway is induced by a variety of growth signals such as insulin, EGF, and calmodulin (upregulated directly by MOR activation), whereby activated PI3K phosphorylates and activates Akt, which can then activate factors such as mTOR [174] and eNOS [175]. Vitamin C has been shown to antagonize these pathways in cancer cells [146,176]. Interactions between opioids and vitamin C on this pathway are further complicated in the ability of morphine to upregulate PTEN [177], a PI3K blocker, while downregulating PP2A phosphorylation, thereby increasing the activity of Akt. Phosphorylated Akt promotes NO and superoxide production from the mitochondrion via dysregulated NADPH oxidases [178,179], leading to further Akt phosphorylation. Generation of free radicals, known to be induced by MOR, can also induce NF-κB and HIF1α translocation to the nucleus [180]. Directly downstream of Akt, mTOR is activated by morphine [181,182,183], yet is repressed by vitamin C; mTOR is central to growth signaling, pathway integration, and numerous degenerative diseases. Inactivation of PP2A by MOR has been shown to increase mTOR signaling [184]. Similar to opposing actions of morphine and vitamin C on mTOR, GSK3β, a negative regulator of glucose metabolism (and therefore oxygen consumption) by Akt, is inactivated (phosphorylated) in the presence of morphine [165], but is activated (via reduced phosphorylation) by vitamin C [185].

Elevated ROS generation, a product of MOR activity [186], can cause NF-κB translocation to the nucleus via modulation of upstream kinases [187], but NF-κB translocation has been shown to be blocked by AA [188]. This transcription factor regulates the expression of a myriad of pro-inflammatory and redox-mediating factors and enzymes; some of these include SOD1 [189], SOD2 [190], Cu/Zn SOD [191], iNOS [192], nNOS [193], NAD(P)H quinone oxidoreductases [194], and even MOR itself [195]. As mentioned previously, Akt can activate NF-κB; Akt activity is linked to morphine signaling [196], but blocked by vitamin C [76]. Translocation of NF-κB is canonically controlled by IKKα/IKKβ heterodimers (with or without a/an IKKγ subunit), which sequester NF-κB into the cytoplasm. Degradation of this IKKα/IKKβ complex via phosphorylation is inhibited by DHA but not AA [197]. The IKKα/IKKβ complex can phosphorylate mTOR [198], further activating it, yet mTOR is repressed by AA. Directly downstream of the IKK complex, IκBα has been shown to be inhibited by AA, as well as NF-κB itself [188]. Synthesizing the known references above leads to the hypothesis that competition of AA:DHA between mTOR and the IKK complex may act as a redox-sensitive switch to control gene regulation by NF-κB, yet this requires further exploration.

HIF1α is a major oxygen sensor and regulator of redox status whose activity is induced by ROS and Akt and inhibited by AA [199], yet has been shown to have mixed effects from opioids [200,201,202,203,204]. Its transcriptional activity leads to the production of antioxidant genes such as iNOS and heme oxygenase-1 [205]. As an upstream cofactor, vitamin C is required for prolyl 3-hydroxylase, prolyl 4-hydroxylases, and asparaginyl hydroxylases to act on HIF1α in order to deactivate its transcriptional activity and ubiquitinate it for degradation [206,207]. HIF1α is also established as a regulator of mitochondrial fatty acid metabolism [208], which is further impacted by aberrant redox signaling due to MOR activation. Both trimethyllysine hydroxylase and γ-butyrobetaine hydroxylase, which utilize vitamin C as a cofactor, are necessary for the synthesis of carnitine that facilitates fatty acid import into the mitochondrion. Morphine has been shown to increase triglyceride content in rat serum, brain [209], and cultured cardiac cells, potentially via triglyceride lipase inhibition [210], yet its effects on individual fatty acids remain to be explored. By combining the known decreases in free fatty acids due to opioid exposure, along with increased concentrations of carnitine from vitamin C, there may be a potential metabolic shift toward oxidative stress in the mitochondria, yet this has not been explored experimentally.

## 8. Conclusions and Future Directions

Opioids are the leading class of drugs of abuse worldwide and continue to rise in popularity while retaining vital roles for analgesia in the clinic. Vitamin C is a well-tolerated exogenous antioxidant with great potential to ameliorate some of the side effects associated with acute and chronic opioid use, but more research must be done to understand the potentially beneficial interactions. While previous research in humans has predominantly focused on integrated physiological responses, such as reductions in pain scores, opioid consumption, and withdrawal symptoms, the underlying mechanisms are poorly understood. The direct antioxidant capacity of vitamin C is one explanation for its effectiveness, but further research is required to optimize dosing and recommended regimens.

Previous studies of vitamin C co-administration have not addressed changes in respiratory parameters, such as blood oxygenation and tidal volume, known to be severely altered by opioids [211,212,213,214]; the impact of vitamin C on potentially fatal OIRD is unknown. These respiratory data are facile and non-invasive to acquire from humans, particularly in clinical settings where they are commonly monitored [215,216]. In animal models, blood oxygenation (SpO2) monitoring is commonplace using pulse oximetry [94,217,218], while plethysmography measurements require more complex instrumentation [219,220,221]. Altered lung function conferred from OIRD may also result in local signaling alterations, particularly in chronic opioid use; animal models are ideal for measuring these tissue changes, as lung biopsy is often not suitable for individuals already undergoing surgery or recovering from chronic opioid use. Illicit use of opioids often involves vaporizing or smoking as well, further implying the need for further investigation into local lung effects.

Further considerations should be made within a local tissue context with regard to metabolism. While many common opioids are prodrugs of active compounds with higher MOR affinity, their metabolites may also have MOR activity, yet these metabolites may be sequestered for excretion before they reach peripheral organs in a sufficient quantity for appreciable activity. For example, morphine-3- and morphine-6-gluconurides, but not morphine, have been implicated in kidney failure [222,223], but excess AA is contraindicated in individuals with kidney disease due to production of oxalate from AA [71]. Hydrophilic metabolites of opioids generated from first-pass metabolism may not sufficiently pass through the blood–brain barrier, thus the route of administration should also be explored as well and given significant consideration, particularly due to the well-established roles of vitamin C in neurotransmission.

The effects of opioids on endogenous antioxidant enzymes have been established, but the role of vitamin C has not been researched extensively. In other oxidative-stress-related conditions and diseases, AA has been shown to have mixed effects on SOD, CAT, and GPx, but these results are difficult to compare due to the types of samples assayed. Animal models are necessary in this context for exploration of tissue and organ-specific effects. Cell-based models may be suitable for these studies, as direct measurements of oxidative stress such as markers of mitochondrial stress (e.g., oxygen consumption and hydrogen ion generation), as well as generation of radical species, can be kinetically monitored.

Individual signaling mechanisms, such as those involving STAT3 and NF-κB, require validation for opioid and AA co-administration. While opioids have been shown to increase, and AA has been shown to decrease, each of these transcription factors’ activities (binding to target genes) is dependent upon upstream signaling from opioids and AA. Therefore, the dose and period of administration, in other words the strength and persistence of upstream signaling, are critical in validating changes in these signaling pathways. Animal models are implicit to define translatable effects of vitamin C and opioid co-administration, yet the presence or absence of *Gulo* and standardization of strain/species used must be taken into account.

Overall vitamin C is safe, relatively non-toxic, inexpensive, and widely available, which justifies it as a potential facile therapeutic or adjuvant for recovery after opioid intoxication. However, much further research is necessary to qualify previous observations of its capacity to ameliorate opioid side effects and define the underlying signaling interactions.

## Data Availability

Data is contained within the article.

## Abbreviations

AA	ascorbic acid
AC	adenylate cyclase
Akt	protein kinase B
aspOHase	asparaginyl hydroxylase
BH3	trihydrobiopterin
BH4	tetrahydrobiopterin
CaM	calmodulin
cAMP	cyclic adenosine monophosphate
CAT	catalase
cNOS	constitutive nitric oxide synthase
CREB	cAMP response element-binding protein
DHA	dehydroascorbic acid
DOR	δ opioid receptor
eNOS	endothelial nitric oxide synthase
ER	endoplasmic reticulum
ERK	extracellular-regulated signaling kinase
GPCR	G-protein-coupled receptor
GPx	glutathione peroxidase
GSK3β	glycogen synthase kinase 3β
Gulo	L-gulonolactone oxidase
HIF1α	hypoxia-induced factor 1α
IKK	IκB kinase
IP3	inositol-3-phosphate
JAK	janus kinase
LC	locus ceruleus
MAPK	mitogen-activated protein kinase
MOR	μ opioid receptor
mTOR	mammalian target of rapamycin
NF-κB	nuclear factor kappa-light-chain-enhancer of activated B cells
nNOS	neuronal nitric oxide synthase
NO	nitric oxide
OIRD	opioid-induced respiratory disorder
ONOO-	peroxynitrite
PI3K	phosphoinositol-3-kinase
PKA	protein kinase A
PLC	phospholipase C
PP2A	protein phosphatase 2A
pro3/4OHase	prolyl 3- and prolyl 4-hydroxylases
RNS	reactive nitrogen species
ROS	reactive oxygen species
SOD	superoxide dismutase
STAT1/2/3	signal transducer and activator of transcription 1/2/3
SVCT1/2	sodium-dependent vitamin C transporters 1/2
TH	tyrosine hydroxylase
TPH	tryptophan hydroxylase
VTA	ventral tegmental area
αi/o	Gαi/o subunit
β/γ	Gβ/γ subunit
